# Dry Cupping and Rest as a Means of Relief in a Case of Locomotor Ataxia

**Published:** 1883-01

**Authors:** Henry M. Lyman

**Affiliations:** Professor of Physiology and of Diseases of the Nervous System, Rush Medical College, Chicago, Ill.


DRY CUPPING AND REST Considered as a Means of Relief in a Case of Locomotor Ataxia. By HENRY M. LYMAN, A.M., M.D., Professor of Physiology and of Diseases of the Nervous System, Rush Medical College, Chicago, Ill.

In the early part of the winter of 1879–80, I was consulted by Mr. D——, a gentleman, then about thirty-eight years of age, a wholesale dry goods salesman, who was suffering with well-marked symptoms of locomotor ataxia. A single man, well-formed and vigorous in his general appearance, he could trace his disorder to no hereditary cause. He was, and always had been, exceedingly temperate and correct in all his habits, and had never experienced any severe sickness. He had never had either syphilis or gonorrhœa, or rheumatism. Shortly after the great Chicago fire in 1871, at which time he had been severely overworked, he began to suffer with lancinating pains and with paroxysms of painful micturition. For this he was treated at great length by an eminent genito-urinary specialist, who considered it a case of spasmodic stricture of the urethra. Treatment with bougies, etc., failed to give any relief, and was finally discontinued. For a number of years the patient continued his usual occupations with the greatest diligence and prosperity, and with the exception of the pains just mentioned he seemed to enjoy excellent health. During the course of the year 1879 he began to experience a certain degree of unsteadiness when walking, especially in the dark. After consulting a number of other physicians, he called at my office, and requested an examination. I found him presenting the usual symptoms of progressive locomotor ataxia—lightning pains, anæsthetic, paresthetic, and analgesic patches upon the surface of the limbs, absence of the tendinous reflexes, ataxic gait, diminution of the power of co-ordination in the muscles of the upper extremities, cracking of the joints, paroxysms of hyperæsthesia affecting the mucous surfaces of the mouth and pharynx, unequal contraction of the pupils, weakened power of accommodation, progressing atrophy of the optic papillæ, and failure of sexual appetite. I advised the use of ergot, which was shortly followed with nitrate of silver, and the daily use of the galvanic current.

Living at a long distance from my office, and actively occupied with the details of his business, the patient was quite inclined to neglect his treatment. He, however, fitted up a galvanic apparatus at home, which he employed with considerable skill. By this means he thought that his pain was somewhat relieved. He could not think of abandoning his work, which was then yielding him a large income, and I soon became convinced that his ataxic symptoms were increasing.

After an absence of several weeks, he one day called upon me, to relate his experience in New York, where he had been obliged to spend some time in connection with his business. While there he had consulted Professor E. C. Seguin, and was for several days under the eye of Dr. Geo. M. Beard. These gentlemen both concurred in the diagnosis of locomotor ataxia, and warned him against a marriage, for which he was making preparations, an opinion with which I heartily agreed.

During the month of February, 1880, my patient was quite tractable, and underwent a course of energetic treatment with the galvanic current, applied long enough over the back every day to produce great redness of the surface. He also continued the use of nitrate of silver. After taking about a drachm of the silver salt, iodide of potassium and bichloride of mercury were substituted in its place, but without any apparent effect.

As the spring and summer of 1880 progressed there was some relief of the painful symptoms. My patient ceased calling at my office, undertook a journey to California, and again visited New York, so that I did not hear from him again until the last of September, or some time in October, 1880, when he informed me that he had determined to run the risks of matrimony. The marriage took place about the middle of October, and the unfortunate bridegroom was so much exhausted by the event that he was obliged to keep his bed the greater part of the time without the slightest attempt or desire to consummate the union. From this time I lost sight of the case.

A few weeks ago I was much surprised by the information that my former patient had been completely cured by the use of Junod's boot in the hands of a non-medical empiric. I at once took measures to ascertain the residence of Mr. D——, and opened communication with him again. From him I learned the following continuation of his history.

Almost simultaneously with the occurrence of his marriage the mercantile house with which he was connected became suddenly and unexpectedly bankrupt. By this failure he found himself thrown out of employment at a time when he was perfectly helpless. His physical condition became rapidly worse. Eyesight began to fail; ataxia increased, and the muscles of the lower extremities began to dwindle, lightning pains were frequent and agonizing. The rectum became anæsthetic and uncertain in its retention of fæces. The feet were cold and numb. When sitting erect the gluteal muscles would seem to roll outward with a sensation as if the tuberosities of the ischia had suddenly slipped down over the edges of the muscles. Walking was becoming very difficult, and was quite impossible in a dark room.

While in this pitiable condition some one advised him to consult an empiric who was treating rheumatism with Junod's boot. The interview did not produce a favorable impression, but inasmuch as the remedy was offered gratuitously if no benefit should accrue, Mr. D—— concluded to make the experiment January 8, 1881. At first there was no apparent benefit, though the apparatus was applied twice every day. After about a month the pains began to diminish, and the circulation began to improve in the extremities. Thus encouraged Mr. D—— procured a set of the cups, etc., and his devoted wife conducted the treatment at home. The cups were applied along the spine, and the limbs were treated with the boot. Gradually improvement took place, and during the summer of 1881 the pains were entirely relieved, and have never returned. The muscles also resumed their former volume; the rectum recovered its sensibility, and the sphincter renewed its appropriate function. Eyesight improved until now he is able to read without the slightest discomfort. Appetite returned; digestion was restored; the power of locomotion increased; and at the end of a year after the commencement of treatment, erections began once more to occur.

On examining Mr. D—— I found everything as he represented it. There is still some degree of ataxia manifest in the movement of the feet, especially when excited or weary. The upper extremities are perfectly manageable. Anæsthesia has disappeared from the regions where it was formerly manifested, but the tendinous reflexes are still absent. The patient walks easily and rapidly without a cane, turning sharply around without difficulty, but he is unsteady on his feet with his eyes shut, and is unwilling to trust himself in the dark. The eyeballs move perfectly, but the pupils are contracted and do not respond readily to changes of light, though they move somewhat in accommodation. There is considerable atrophy of the optic disk. Difficulty is apparent in recalling proper names, but otherwise the intellectual processes are intact. Appetite, digestion, and sleep are perfectly natural. The patient no longer feels that excessive sensibility to changes of temperature which formerly troubled him. Erections take place as often and as completely as ever. Amatory feelings have revived, and sexual intercourse has been performed several times without much subsequent lassitude, but for prudential reasons has been discontinued. The patient feels competent to resume business, but is wise enough to see that his disease is not cured, and that discretion is still the better part of valor. He still continues the use of the cupping apparatus every other day, and is positive in his conviction that his improvement, though gradual is progressive. It is certainly true that his condition is vastly superior to what it was when I previously saw him at the time of his marriage—an improvement which is due in part to his long vacation from business, and in no inconsiderable part to the powerful counter irritation and derivation effected by the use of the cupping apparatus. If this had produced no better result than the mere relief of pain it would still be invaluable in his estimation. Its effect upon the general health and nutrition gives it the right, in this case at least, to rank with hydrotherapy, massage, and the Swedish movement cure as a method of treatment which deserves trial as a means of retarding the progress and relieving the anguish of one of the most intractable of diseases.

September 8, 1882.

